# LncRNA expression profiling of BMSCs in osteonecrosis of the femoral head associated with increased adipogenic and decreased osteogenic differentiation

**DOI:** 10.1038/s41598-018-27501-2

**Published:** 2018-06-14

**Authors:** Qingyu Wang, Qiwei Yang, Gaoyang Chen, Zhenwu Du, Ming Ren, Ao Wang, Haiyue Zhao, Zhaoyan Li, Guizhen Zhang, Yang Song

**Affiliations:** 1 Department of Orthopedics of the Second Hospital of Jilin University, Ziqiang Street 218, Changchun, Jilin 130041 China; 2 Research Centre of the Second Hospital of Jilin University, Ziqiang Street 218, Changchun, Jilin 130041 China; 3 The Engineering Research Centre of Molecular Diagnosis and Cell Treatment for Metabolic Bone Diseases of Jilin Province, Ziqiang Street 218, Changchun, Jilin 130041 China

## Abstract

Long noncoding RNAs (lncRNAs) are critical gene expression regulators and are involved in several bone diseases. To explore the potential roles of lncRNAs in osteonecrosis of the femoral head (ONFH), we investigated for the first time the lncRNA expression profile of bone marrow mesenchymal stem cells (BMSCs) from patients with steroid-induced ONFH (SONFH) with microarray and bioinformatics analysis. A total of 1878 lncRNAs and 838 mRNAs were significantly up-regulated while 1842 lncRNAs and 1937 mRNAs were statistically down-regulated in the SONFH group compared with control group. The results validated by qRT-PCR were consistent with the microarray profiling data, especially involved in upregulation and downregulation of critical lncRNAs as well as mRNAs expression related to adipogenic and osteogenic differentiation. Pathway analyses revealed 40 signaling pathways with significant differences, especially the signaling pathways to regulate stem cell pluripotency. The CNC and ceRNA network indicated that lncRNA RP1-193H18.2, MALAT1 and HOTAIR were associated with abnormal osteogenic and adipogenic differentiation of BMSCs in the patients with SONFH. Our results suggest the lncRNA expression profiles were closely associated with the abnormal adipogenic and osteogenic transdifferentiation of BMSCs during the development of SONFH and explore a new insight into the molecular mechanisms of SONFH.

## Introduction

Osteonecrosis of the femoral head (ONFH) is a multifactorial and disabling disease that involves multiple genetic and environmental factors^1,2^. The incidence of ONFH has increased over the past decade. It is estimated that 20000 to 30000 new patients are diagnosed with ONFH annually in the United States and 150000 to 200000 new cases in China^3,4^. Moreover, the treatment of adult ONFH, with 8.12 million patients in China, remains a challenge to surgeons^5^. Lipid metabolism disorder has been regarded as a primary factor of ONFH pathogenesis because steroid- and alcohol-induced ONFH are highly relevant to lipid metabolism disorder and excessive fat accumulation in the lesion of bone marrow cavity, concurrent with the increased serum lipid level^6^. Excess steroid use has been recognized as one of environmental risk factors and steroid-induced osteonecrosis of the femoral head (SONFH) is also considered as a disease of abnormal BMSC transdifferentiation due to the roles of steroid in promotion of adipogenic differentiation and inhibition of osteogenic differentiation of BMSCs^7,8^. However, the molecular pathogenesis of SONFH remains unclear.

Long noncoding RNAs (lncRNAs) are a newly discovered class of regulatory molecules that affect a variety of biological processes involved in cell differentiation and gene expression regulation^9^. lncRNAs function as competing endogenous RNAs (ceRNAs), which compete for binding to microRNAs to regulate gene expression^10^. Recent studies have revealed the involvement of lncRNAs in regulation of osteogenic differentiation of BMSCs^11,12^. Furthermore, abnormal expression of lncRNAs influences the osteogenic differentiation of BMSCs, which leads to the development of orthopedic diseases^13,14^. However, the biological role of lncRNA expression profiles in the abnormal adipogenic and osteogenic transdifferentiation of BMSCs in ONFH has not been reported. Here, we investigated lncRNAs expression profiles in the patients with SONFH and their association with the imbalanced osteogenic and adipogenic differentiation of BMSCs to explore the potential roles of lncRNAs expression in the molecular mechanisms of SONFH.

## Results

### The phenotypes, proliferation, and cell cycle of BMSCs showed no significant differences between SONFH and control groups

The BMSCs from SONFH and control groups were all observed, and all exhibited the morphologically characteristics of plastic-adherent and spindle-shaped cells (Supplementary Fig. 1A). There were no significant differences in the proliferative rates between SONFH and control groups (Supplementary Fig. 1B). The BMSCs had typical mesenchymal stem cells surface markers, positive for CD90, CD73, and CD105 and negative for CD34 and CD45 (Supplementary Fig. 1C). The cell cycle distribution of BMSCs in both groups was not statistically different (Supplementary Fig. 1D,E).

### Osteogenic differentiation of BMSCs in the SONFH group was decreased

**Figure 1:**
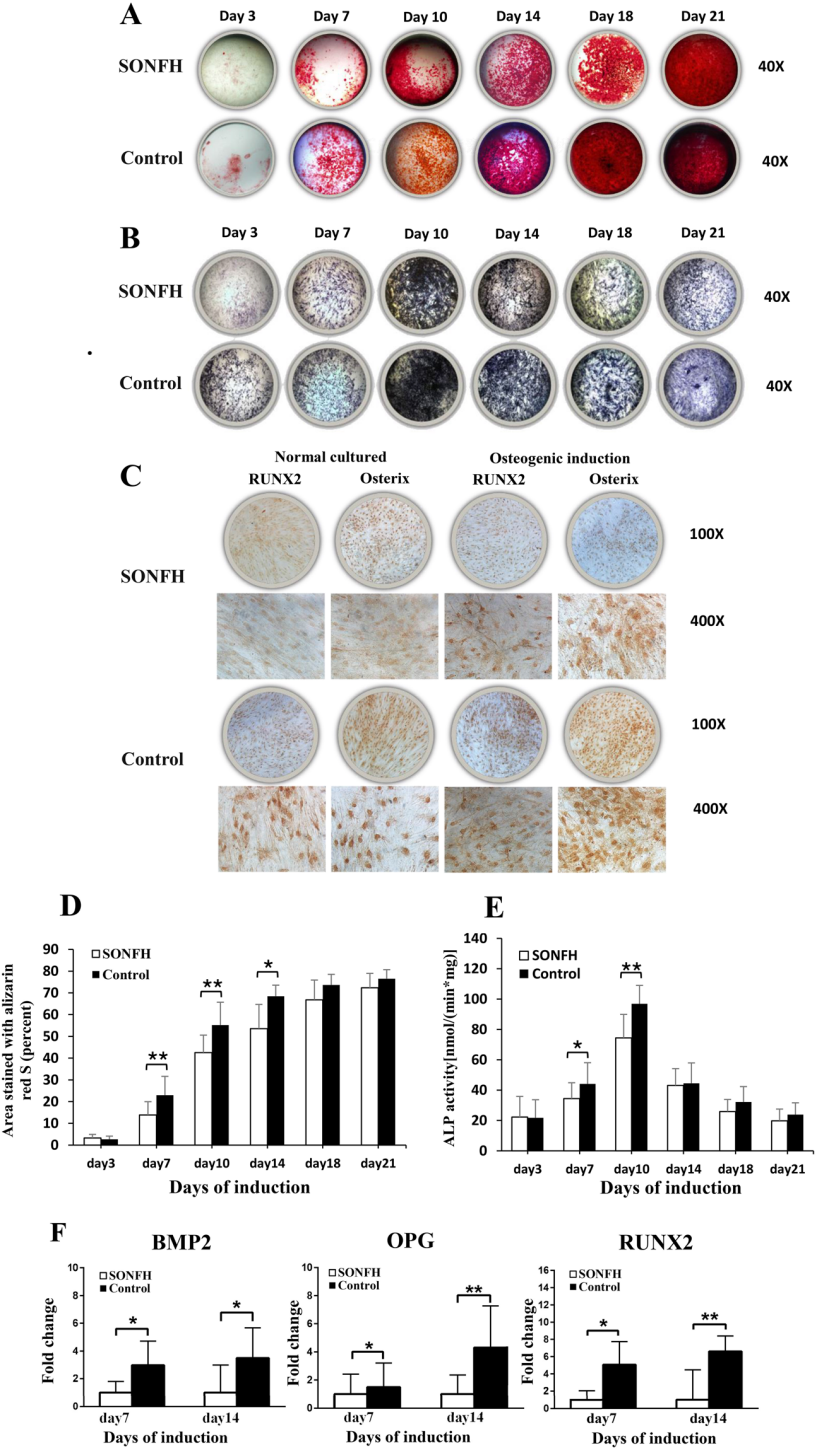
The osteogenic differentiation ability of BMSCs between the SONFH (n = 16) and control groups (n = 16). The SONFH group had weaker ARS (**A**) and ALP (**B**) staining (40×) than the control group. (**C**) The SONFH group showed weaker RUNX2 and Osterix immunohistochemical staining. (**D**) The proportion of ARS-stained areas quantified in the SONFH group was significantly lower than that of control group at day 7, 10 and 14. (**E**) The ALP activity of the SONFH group was significantly decreased compared to that of the control group at days 7 and 10. (**F**) The expression levels of BMP2, OPG and RUNX2 during osteogenic differentiation of BMSCs in the SONFH group were significantly decreased comparing to the control group. All samples were normalized to the expression of GAPDH, and the relative expression levels of each gene were analyzed using the 2^−△△Ct^ method. *P < 0.05, **P < 0.01.

After the BMSCs from both groups were cultured in osteogenic medium for 21 days, ARS, ALP staining and immunohistochemical staining of osteogenic markers including Runt related transcription factor 2 (RUNX2) and Osterix were used to determine the osteogenic differentiation ability of the cells. The results showed that BMSCs in the SONFH group had weaker ARS and ALP staining compared to control group (Fig. 1A,B). BMSCs from the SONFH group had weaker RUNX2 and Osterix immunohistochemical staining both before and after osteogenic induction compared to the control group (Fig. 1C). At day 7, 10 and 14 after osteogenic induction, the ARS quantification of the SONFH group was significantly lower than that of the control group (Fig. 1D). The ALP activity of both groups was peaked on day 10, and the activity in the SONFH group was significantly lower than that in the control group at day 7 and day 10 (Fig. 1E). Additionally, we analyzed gene expression in both groups during osteogenic differentiation using qRT-PCR. The results showed that osteoblastic markers including bone morphogenetic protein 2 (BMP2), osteoprotegerin (OPG) and RUNX2, were expressed at significantly lower levels during osteogenic differentiation of BMSCs from the SONFH group than those from the control group [P < 0.05, Fig. 1(F)].

### Adipogenic differentiation of BMSCs in the SONFH group was increased

**Figure 2:**
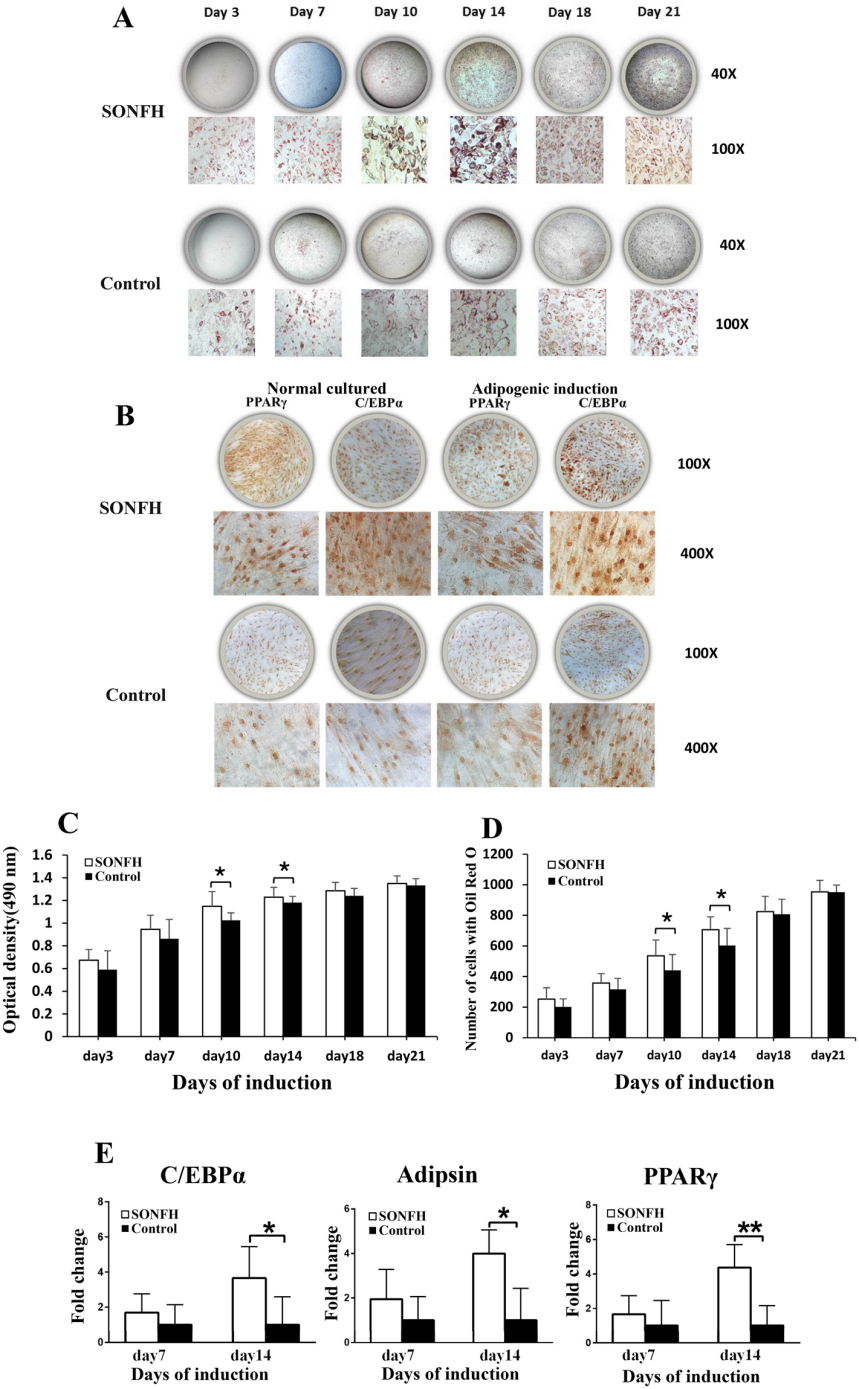
The adipogenic differentiation ability of BMSCs between the SONFH (n = 16) and control groups (n = 16). (**A**) The SONFH group had stronger Oil Red O staining (40× and 200×) than the control group. (**B**) For immunohistochemical staining of adipogenic markers (PPARγ, C/EBPα), the SONFH group had stronger staining than the control group. (**C**) The Oil Red O quantification of SONFH group was significantly higher than that of the control group at day 10 and 14. (**D**) The number of cells with Oil Red O staining (100×) in SONFH group was significantly higher than control group at day10 and day 14. (**E**) The expression levels of PPARγ, C/EBPα and Adipsin during adipogenic differentiation of BMSCs in the SONFH group were significantly increased comparing to the control group. All samples were normalized to the expression of GAPDH, and the relative expression levels of each gene were analyzed using the 2^−△△Ct^ method. *P < 0.05, **P < 0.01.

After the BMSCs of both groups were cultured in adipogenic medium for 21 days, Oil Red O staining and immunohistochemical staining of adipogenic markers including peroxisome proliferator activated receptor gamma (PPARγ) and CCAAT/enhancer binding protein α (C/EBPα) staining were used to assess the adipogenic differentiation potential of the cells. The results showed that BMSCs in the SONFH group had stronger Oil Red O staining than did cells in the control group (Fig. 2A). BMSCs from the SONFH group had stronger PPARγ and C/EBPα immunohistochemical staining both before and after adipogenic induction compared to the control group (Fig. 2B). At day 10 and 14 after adipogenic induction, the Oil Red O quantification of ONFH group was significantly higher than that of the control group. (Fig. 2C). The number of cells with Oil Red O staining in SONFH group was significantly higher than control group at day 10 and 14 (Fig. 2D). For adipogenic markers, including PPARγ, C/EBPα and Adipsin, BMSCs from the SONFH group tended to have significantly higher expressions than those from the control group (P < 0.05) (Fig. 2E).

### Differential expression profiles of lncRNAs and mRNAs in BMSCs from the SONFH and control groups

**Figure 3:**
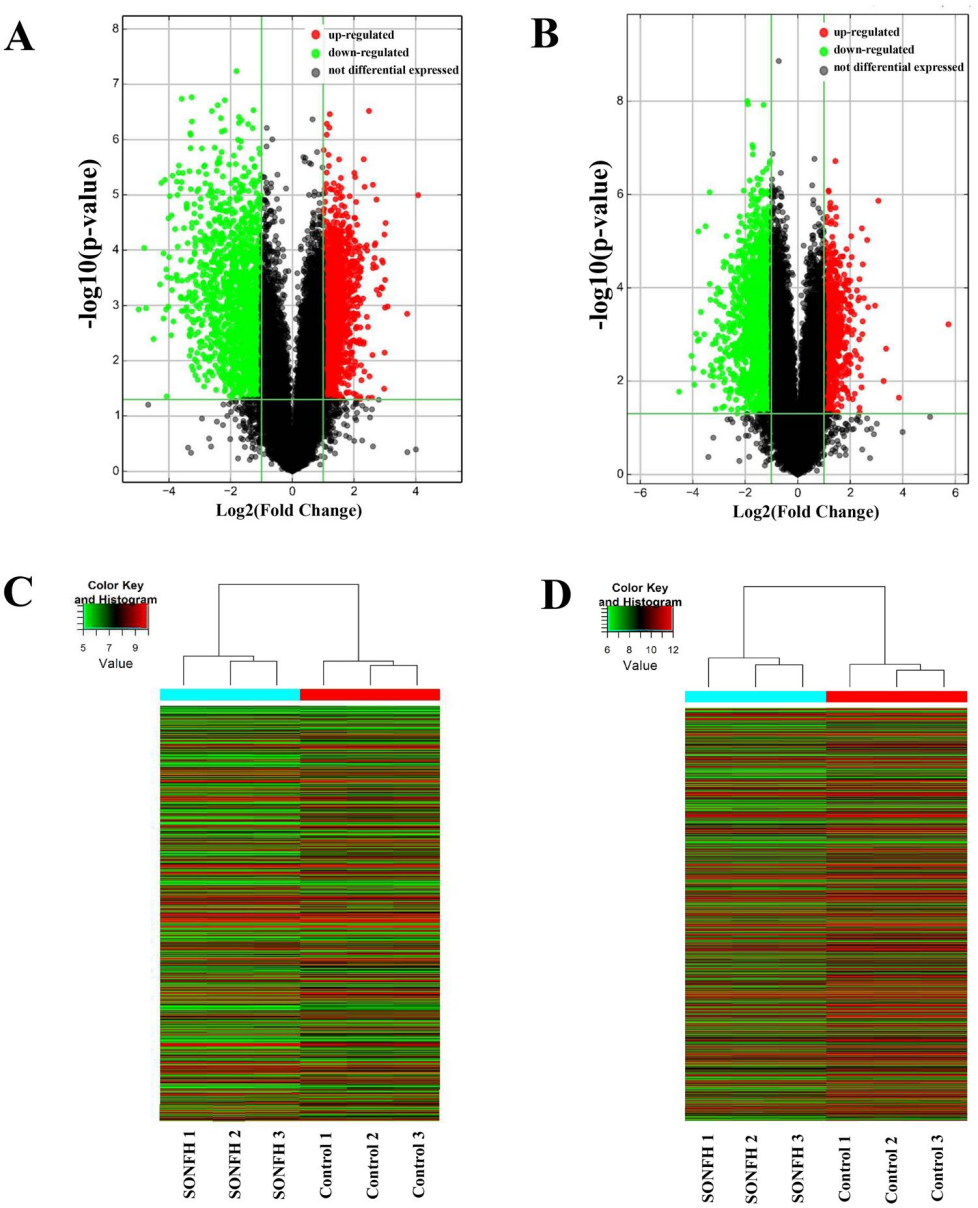
Overview of aberrantly expressed lncRNAs and mRNAs in SONFH. (**A,B**) The Volcano Plot of lncRNA and mRNA expression. Each point represented one lncRNA or one mRNA. The red points (up-regulated) and green points (down-regulated) indicated a change in lncRNA or mRNA expression of more than 2.0-fold. (**C,D**) Hierarchical clustering showed a distinguishable lncRNA or mRNA expression profile between the two groups and homogeneity within groups. Each row represented an lncRNA or mRNA and each column represented a sample. Red color represented a high relative expression level; green color represented a low relative expression levels. RNA was extracted from the BMSCs of three SONFH patients and three control patients.

Volcano plots revealed that lncRNAs and mRNAs were differentially expressed between SONFH patients and control subjects (Fig. 3A,B). Hierarchical clustering indicated that the lncRNA and mRNA expression profiles were distinguishable between the two groups (Fig. 3C,D). Principal Component Analysis (PCA) showed that different samples in the same experiment group were arranged in a compact and clustered manner which indicated good repeatability. The different experimental groups are separated from each other to make a clear division and showed good specificity (Supplemental Fig. 2A,B). A total of 3720 lncRNAs and 2775 mRNAs were differentially expressed in SONFH group BMSCs compared with control group BMSCs (Supplementary Table 1). In total, 1878 lncRNAs and 838 mRNAs were up-regulated, and 1842 lncRNAs and 1937 mRNAs were down-regulated (fold change >2.0, P value < 0.05). The top ten up- and down-regulated lncRNAs are listed in Table 1.

**Table 1:** The top ten up- and down-regulated lncRNAs between SONFH group and control group.

lncRNAs	Gene Symbol	Regulation	Fold change	Chromosome	Strand	RNA length
T189748	G043636	Up	16.9349329	chr2	−	1752
NR_102430	OGFR-AS1	Up	13.2261262	chr20	−	684
T306101	G071509	Up	8.5383133	chr6	+	475
T224056	G051725	Up	8.1545476	chr21	+	32113
TCONS_00018332	XLOC_008610	Up	8.0527711	chr10	+	368
NR_038340	LOC100505817	Up	8.0369531	chr18	+	1181
uc.87	uc.87	Up	7.9673451	chr2	−	290
uc003bpu.1	BC141932	Up	7.9399488	chr3	−	596
ENST00000428667	AP000695.4	Up	7.8981161	chr21	+	715
ENST00000563515	RP11-356C4.5	Up	7.6740573	chr16	−	416
ENST00000588041	RP1-193H18.2	Down	31.5558738	chr17	+	2504
ENST00000606654	LL22NC03-2H8.5	Down	27.8118087	chr22	−	3275
ENST00000607490	XXbac-BPGBPG55C20.3	Down	26.7325588	chr6	−	540
ENST00000517884	RP11-662G23.1	Down	22.5294148	chr8	−	833
T083105	G019270	Down	19.5250793	chr12	+	731
ENST00000605586	RP1-142L7.8	Down	18.9465697	chr6	−	594
ENST00000518507	RP11-110G21.2	Down	18.0318677	chr8	−	283
ENST00000425296	THRB-IT1	Down	18.0209759	chr3	−	1754
NR_026813	LINC00597	Down	17.4490437	chr15	−	1512
uc003ktd.3	BC043373	Down	17.0209879	chr5	−	1651

### Validation of differentially expressed lncRNAs and mRNAs by qRT-PCR

**Figure 4:**
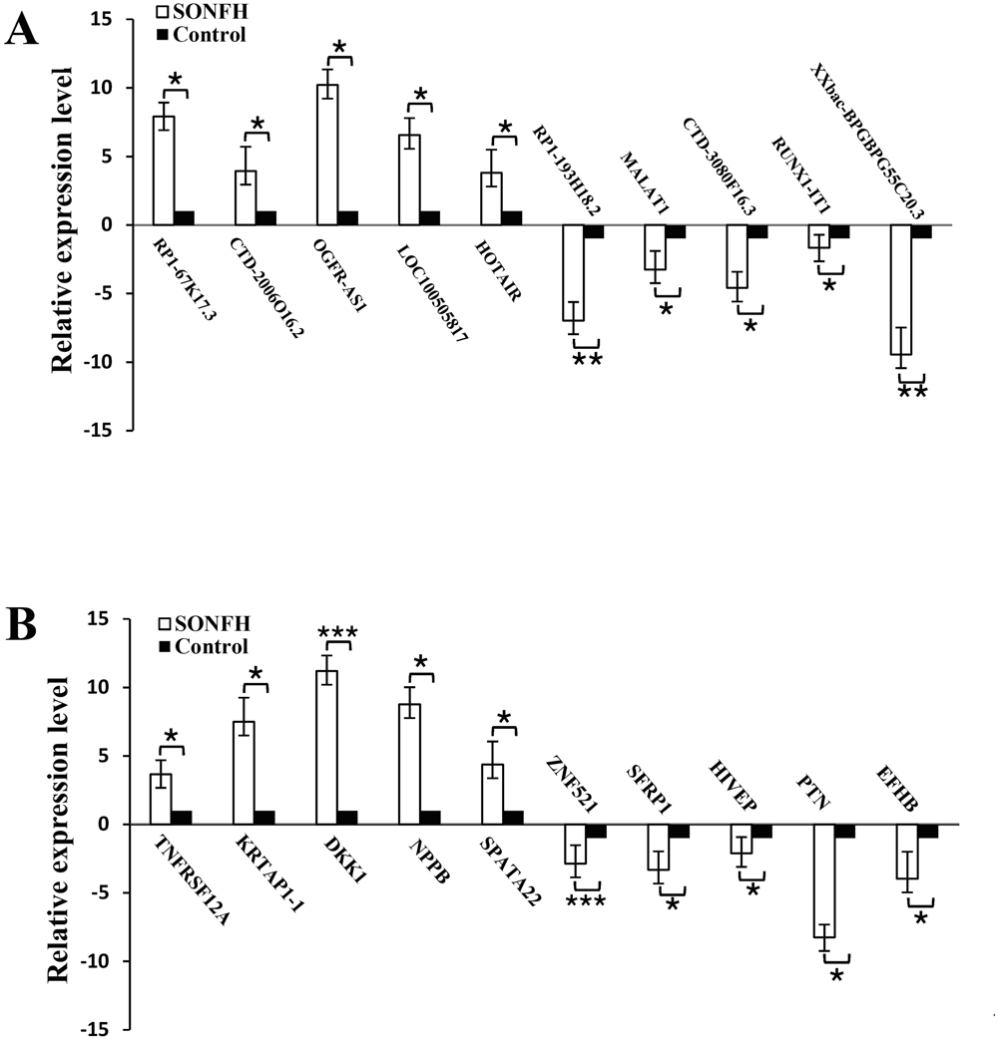
Validation of the lncRNA and mRNA microarray results by real-time qPCR. LncRNA(**A**) and mRNA(**B**) microarray results were verified by real-time qPCR between the SONFH group (n = 12) and the control group (n = 12). All samples were normalized to the expression of GAPDH, and the relative expression levels of each gene were analyzed using the 2^−△△Ct^ method. *P < 0.05, **P < 0.01 and ***P < 0.001.

To confirm the reliability of the microarray data, we selected 10 differentially expressed mRNAs and 10 lncRNAs (5 up-regulated and 5 down-regulated) and measured their expression by qRT-PCR (Supplementary Table 2). Consistent with the microarray data, the 5 up-regulated lncRNAs and mRNAs were OGFR-AS1, LOC100505817, HOTAIR, RP1-67K17.3, and CTD-2006O16.2 (5 lncRNAs), and KRTAP1-1, TNFRSF12A, DKK1, NPPB, and SPATA22 (5 mRNAs). The 5 down-regulated lncRNAs and mRNAs were RP1-193H18.2, XXBAC-BPGBPG55C20.3, MALAT1, CTD-3080F16.3, and RUNX1-IT1 (5 lncRNAs) and PTN, SFRP1, ZNF521, HIVEP, and EFHB (5 mRNAs), respectively (Fig. 4A,B).

### Bioinformatics analysis of differentially expressed lncRNAs and mRNAs

**Figure 5:**
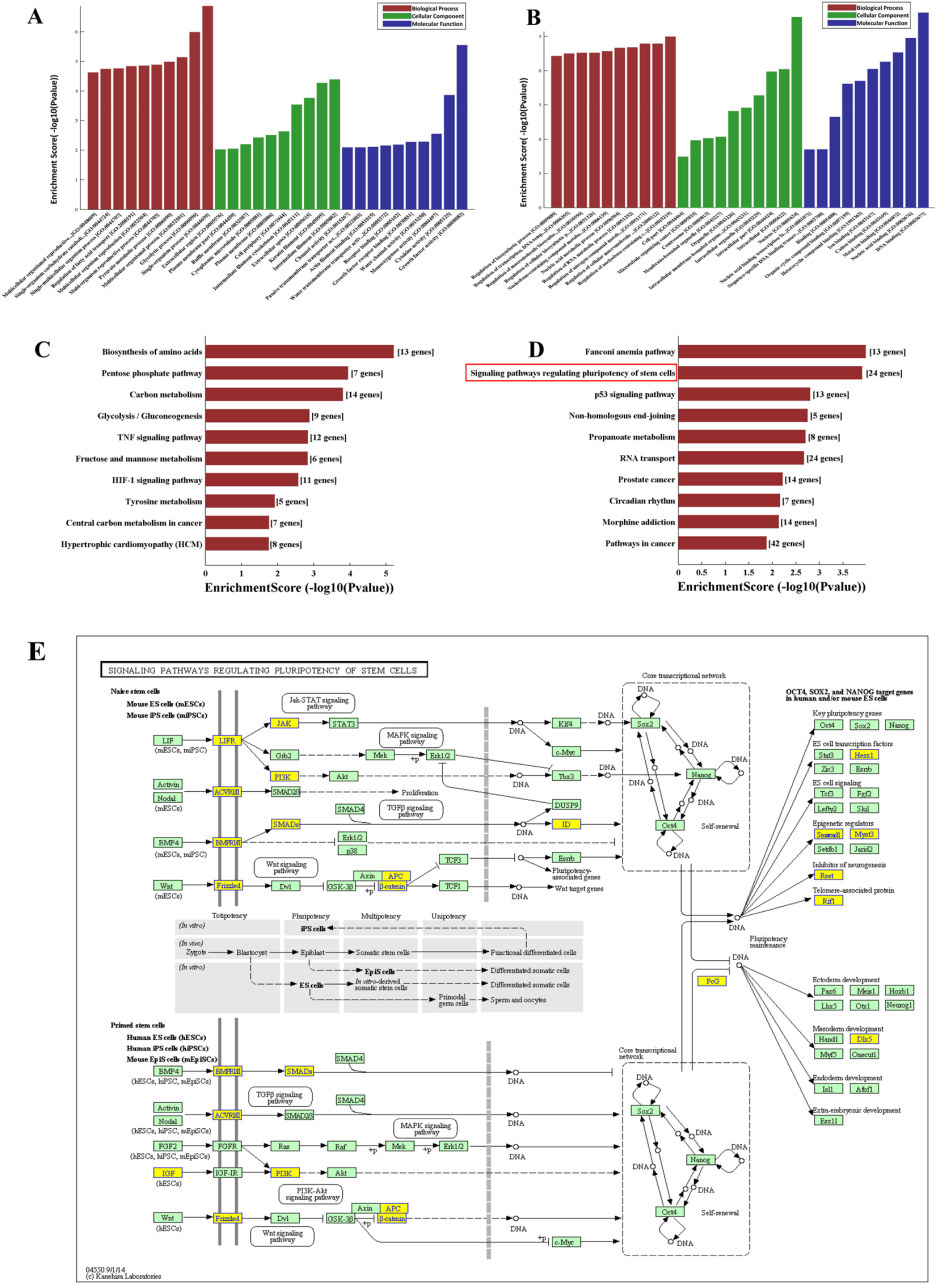
GO and pathway analysis of differentially expressed mRNAs. The GO analysis covered the three domains biological process, cellular component and molecular function. (**A**,**B**) Enriched up-regulated and down-regulated GO terms for each of the categories. (**C**,**D**) Significantly up-regulated and down-regulated pathways. (**E**) The signaling pathways regulating the pluripotency of stem cells. A total of 24 differentially expressed gene were enrich in this pathway category which included Jak-STAT, MAPK, TGFβ, Wnt and PI3K- Akt signaling pathways. The genes in the yellow boxes were down-regulated and the genes in the green boxes showed no change in expression.

GO analysis was used to analyze the main functions of the differentially expressed genes according to gene ontology which included biological processes, cellular components and molecular functions. Single organism process, intermediate filament and growth factor activity were the most significantly up-regulated terms enriched in each of the three categories (Fig. 5A). Regulation of nucleobase-containing compound metabolic process, nucleus and DNA binding were the most significantly down-regulated terms enriched in each of the three categories (Fig. 5B). The KEGG database was used to confirm the pathways mediating the functions of the differentially expressed genes. Forty pathways showed significant differences due to differential gene expression, including 16 pathways involving up-regulated genes, and 24 pathways involving down-regulated genes (Fig. 5C,D). The significantly up-regulated pathways consisted of biosynthesis of amino acids, the pentose phosphate pathway and carbon metabolism (P < 0.05 and Enrichment Score >3). Pathway analysis showed that the significantly down-regulated pathways consisted of the signaling pathways regulating the pluripotency of stem cells (P < 0.05 and Enrichment Score >3). The altered genes related to signaling pathways that regulated the pluripotency of stem cells are shown in Fig. 5E. All results of GO and pathway analysis are shown in Supplementary Table 3.

**Figure 6:**
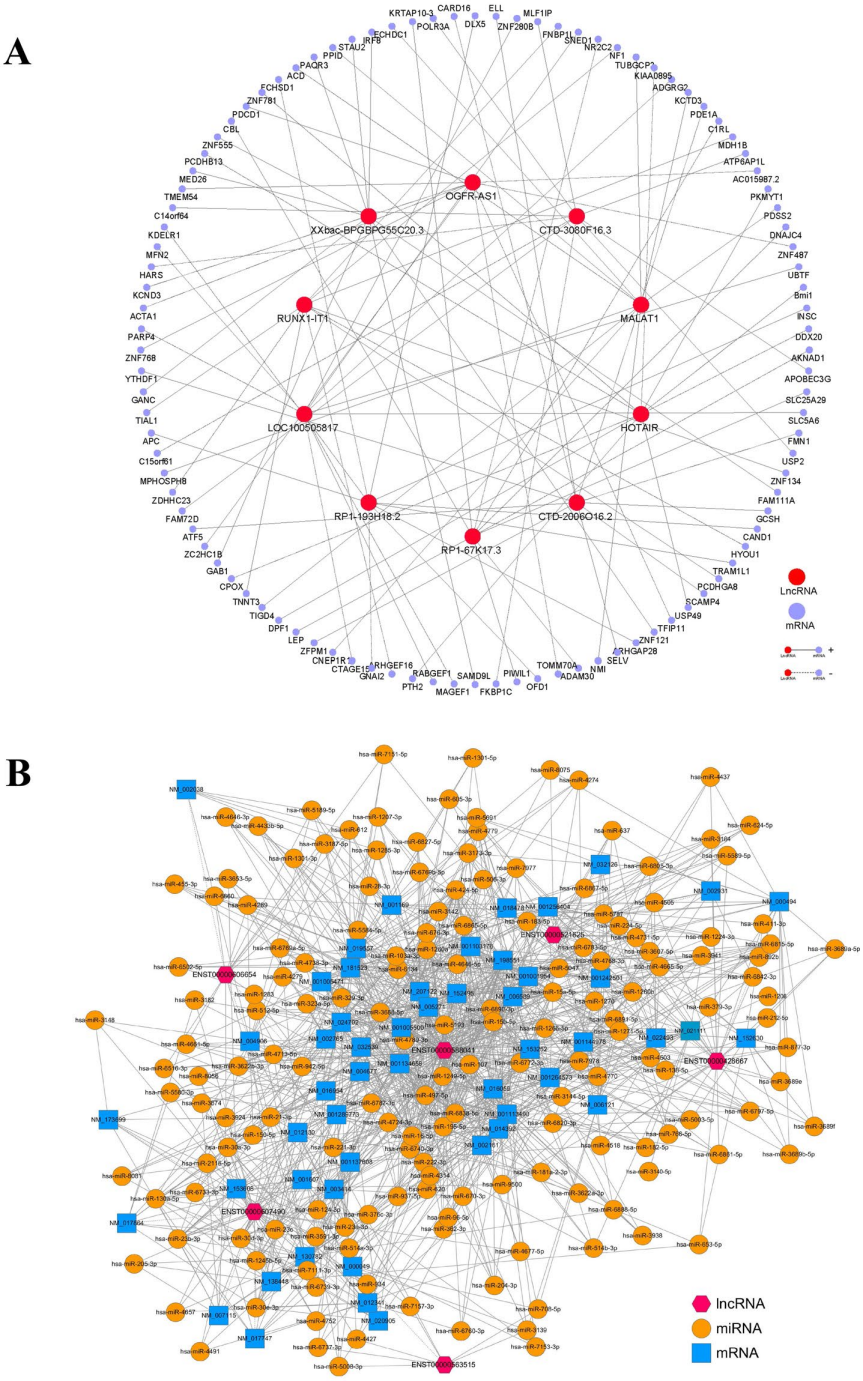
The CNC and ceRNA network. (**A**) For the CNC network, the lines represented the regulatory relationships between genes (solid lines represened positive correlations, dotted lines represened negative correlations). Nodes in light blue were mRNAs. Nodes in red were lncRNAs. (**B**) For the ceRNA network, nodes in yellow were microRNAs. Nodes in light blue were mRNAs. Nodes in magenta were lncRNAs.

A total of 10 lncRNAs and 100 mRNAs were used to construct a CNC network to ascertain correlations between differentially expressed lncRNAs and mRNAs (Fig. 6A). In the CNC network, each mRNA can be correlated with one to tens of lncRNAs and vice versa. The lncRNAs HOTAIR and RP1-193H18.2 had substantially more associated mRNA that were related to osteogenic and adipogenic differentiation of BMSCs than the other lncRNAs. The CNC network was used to determine the inter-regulation of lncRNAs and mRNAs in the molecular mechanisms underlying the different differentiation ability of BMSCs from SONFH patients. Moreover, a lncRNA-miRNA-mRNA ceRNA network was also constructed which included 6 lncRNAs, 170 miRNAs, and 52 mRNAs (Fig. 6B). The ceRNA network shows lncRNAs can act as miRNA “sponges” which inhibit interaction with their miRNA targets in post-transcriptional regulation.

## Discussion

ONFH is considered as a stem cell disease, which is associated with imbalance osteogenesis and adipogenesis differentiation of BMSCs, and the upregulation of adipogenic differentiation may play an essential role on the lipopexia of ONFH^15^. Osteoblasts and adipocytes are originated from a common progenitor cell of BMSCs. Adipogenesis induction factors has inhibitory effect on osteogenesis, whereas osteogenesis induction factors suppress adipogenesis^16,17^. The transdifferentiation between osteogenesis and adipogenesis differentiation of BMSCs contains different transcription factors and signaling pathways^18^. Some results have well demonstrated that proliferative capability of BMSCs in ONFH was significantly decreased^8^. For example, the replicative capacity of osteoblastic cells from BMSCs in ONFH patients was reduced compared with patients with osteoarthritis^19^, and the BMSC numbers and proliferative capability are depressed in patients with ONFH^20^. Moreover, the activities of BMSCs and the number of Fibroblast colony-forming units (CFU-Fs) from the iliac crest were decreased during isolation culture in patients with idiopathic, alcohol-induced or steroid-induced ONFH than in the control group^21^. Moreover, the CFU-Fs number in the proximal femur of patients with steroid-induced ONFH was also found to be decreased^22^. Another study reported that BMSCs obtained from proximal femur marrow of patients with alcohol-induced ONFH had lower osteogenic differentiation ability and doubling time than cells obtained from patients with femoral neck fracture^23^.

However, our results failed to show a significant difference in the morphology, proliferation, and phenotypes of BMSCs in primary and passaged cultures between the SONFH group and the control group, which was consistent with previous studies that showed no significant reduction in the proliferative activities of BMSCs of the patients with ONFH during BMSCs differentiation *in vitro*^8,24^. As previously reported, BMSCs isolated from fresh bone marrow of the iliac crest were differentiated into an osteogenic lineage, and the proliferative activity as well as their osteogenic ability of BMSCs from patients with ONFH were unaltered during differentiation *in vitro*. Moreover, results from patients with idiopathic or alcohol-induced ONFH were similar to those of matched patients with non-necrotic disorder^24^. These contrasting results may relate to that (I) BMSC activities were not measured when ONFH occurred, and most of the subjects had late-stage disease and showed joint destruction when the bone marrow was aspirated; (II) the time between disease occurrence and marrow cell harvest might permit recovery of abnormal bone marrow function; (III) isolation yield and proliferation capacity may be dependent on donor-specific variables^25,26^.

More important, the osteogenic and adipogenic differentiation ability of BMSCs from patients with SONFH were significantly decreased and increased, respectively, in this study. Specifically, the increased expression of BMP2, OPG, RUNX2 and decreased expression of PPARγ, C/EBPα, Adipsin were observed in the SONFH group comparing to the control group. PPARγ is an adipogenic transcription factor which plays an important role in promoting adipogenic differentiation and inhibiting osteogenic differentiation^26,27^. Previous results have demonstrated that steroids up-regulate PPARγ expression in rodent and human BMSCs and lead to time- and dose-dependent induction of adipogenic differentiation^1,28^. Excessive fat accumulation has been observed in the lesion area of bone marrow cavity and lipid metabolism disorder has been regarded as a critical factor of ONFH. The expression level of BMP2, RUNX2 and Osterix were significantly decreased and PPARγ expression was significantly increased in the lesion area of SONFH^29,30^. All these findings suggest critical roles of the imbalance between adipogenic and osteogenic transdifferentiation of BMSCs in the occurrence and development SONFH.

Multiple genetic and environmental factors are involved in the development process of ONFH, especially, steroids are one of the environmental factor which have major effects on the differentiation of stem cells. As it’s shown in the immunohistochemistry staining, weaker RUNX2, Osterix staining and stronger PPARγ and C/EBPα staining were observed in BMSCs from the SONFH group no matter before or after osteogenic induction (Fig. 1C,B). However, the basal expression levels of osteogenic markers (RUNX2, Osterix, BMP2, OPG) in BMSCs without osteogenic induction showed no significant difference between these two groups (Supplemental Fig. 3). Therefore, we deduced that the potential differentiation ability of BMSCs has been affected by the stimulation of steroids. Although the basal expression levels of osteogenic markers in BMSCs without osteogenic induction showed no significant difference between SONFH and control groups, significantly increased adipogenic and decreased osteogenic differentiation capacity were confirmed when BMSCs were stimulated by excess steroids.

LncRNAs have been investigated in several bone disease including ankylosing spondylitis^14^, knee osteoarthritis^31^, and postmenopausal osteoporosis^32^, but the lncRNA expression profiles of ONFH have never been reported. In this study, for the first time we completed the lncRNA expression profiles of BMSCs in SONFH and found that 1878 lncRNAs and 838 mRNAs were significantly up-regulated while 1842 lncRNAs and 1937 mRNAs were statistically down-regulated in the SONFH group, compared with the control group. The results validated via qRT-PCR were also consistent with the lncRNA and mRNA expression profile data, especially involved in critical lncRNAs and mRNAs expression of adipogenic and osteogenic differentiation. Pathway analysis revealed that 40 pathways exhibited significant differences in gene expression, including 16 pathways involving up-regulated genes, and 24 pathways involving down-regulated genes. All of the significantly up-regulated pathways pertained to biosynthesis of amino acids, the pentose phosphate pathway and carbon metabolism while the significantly down-regulated pathways were involved in regulating the pluripotency of stem cells.

The signaling pathways that regulate the pluripotency of stem cells may play a significant role in the molecular pathogenesis of SONFH because the occurrence and development of ONFH is closely associated with the imbalance transdifferentiation between osteogenesis and adipogenesis of BMSCs. Our results also showed that adenomatous polyposis coli (APC) and distal-less homeobox 5 (DLX5) in signaling pathways that regulate the pluripotency of stem cells were significantly down-regulated. In addition, a CNC network was constructed using 10 lncRNAs and 100 mRNAs to ascertain correlations between differentially expressed lncRNAs and mRNAs of BMSCs. In the CNC network, APC and DLX5 expression was found to be significantly correlated with RP1-193H18.2 (Pearson correlation coefficient ≥0.99, FDR ≤ 0.05), which was the most down-regulated lncRNA (fold change, 31.56) in our microarray results. Previous research has shown that APC knockdown can block osteogenic differentiation of skeletal progenitor cells^33^, and miR-142-3p promotes Wnt signaling through APC inhibition^34^. DLX5 is a bone-inducing transcription factor and is associated with osteoblast differentiation^35^. These findings revealed for the first time RP1-193H18.2 plays a potential role in down-regulated BMSC osteogenic differentiation.

Homeobox transcript antisense RNA (HOTAIR) was first discovered in 2007 and has been shown to be correlated with disease progression in patients with thyroid cancer, bladder cancer, Parkinson’s disease, and esophageal cancer^36–39^. In our study the expression level of HOTAIR (lncRNA) was significantly higher in the SONFH group than the control group. HOTAIR has been reported to inhibit miR-17-5p to regulate osteogenic differentiation and proliferation in non-traumatic ONFH^40^. Our CNC network showed that HOTAIR was significantly correlated with Bmi1 (Pearson correlation coefficient ≤ −0.99, FDR ≤ 0.05). Bmi1 is a member of the PcG (Polycomb group) family of epigenetic regulators, and overexpression of Bmi1 can stimulate skeletogenesis by improving the osteogenic microenvironment^41^. Bmi1 was also down-regulated (fold change, 2.11) in our mRNA microarray data. The relationship between HOTAIR and Bmi1 may be a novel pathogenic mechanism in the abnormal differentiation of BMSCs in SONFH and merits further investigation.

More than 10,000 lncRNAs have been found to function as potential ceRNAs^42^. Previous studies have demonstrated that lncRNAs can serve as miRNA “sponges” that inhibit interaction with their miRNA targets in post-transcriptional regulation. Most of these studies have focused on liver cancer, lung cancer and other malignant tumors involved in regulation of cell cycle and cell death^43,44^. However, few studies have reported the involvement of ceRNA interactions in BMSC differentiation in SONFH. The ceRNA network constructed in our study revealed that RP1-193H18.2 acted as an endogenous sponge of several miRNAs to down-regulate RECK (reversion-inducing cysteine-rich protein with Kazal motifs, NM_021111). RECK has been identified as a master switch between osteogenic and adipogenic differentiation. RECK depletion reduced the capacity of mesenchymal stem cells to differentiate into the osteogenic lineage, whereas adipogenesis was increased^45^. In future studies, the correlation between RP1-193H18.2 and RECK and their potential roles in the increased adipogenesis observed in SONFH should be explored.

Metastasis-associated lung adenocarcinoma transcript 1 (MALAT1) is frequently overexpressed in malignant tumors and is considered as a biomarker for a variety of tumors. A previous study demonstrated that MALAT1 promoted osteogenic differentiation of aortic valve interstitial cells by sponging miR-204^46^. Our results showed that MALAT1 was significantly down-regulated, indicating a possible role in the decreased osteogenic differentiation of BMSCs in SONFH patients. Adipogenesis activation requires suppression of the canonical Wnt signaling pathway^47^. The dickkopf protein encoded by dickkopf Wnt signaling pathway inhibitor 1 (DKK1) is an antagonistic inhibitor of the Wnt signaling pathway^48^. Our results showed that the DKK1 expression level in BMSCs from SONFH patients was significantly increased (P < 0.001). This finding revealed that abnormal expression of DKK1 played a significant role in the progression of adipogenesis and suppression of osteogenic differentiation in SONFH.

Our study has several limitations. First, our BMSC sample size for microarray analysis was small which may influence the lncRNAs and mRNAs expression profiles data. Second, most of the differentially expressed lncRNAs and mRNAs still need to be validated. Finally, although the CNC network and ceRNA network of lncRNAs and mRNAs were constructed in the present study, the mechanisms of these lncRNAs and mRNAs need to be confirmed in further specific studies.

In summary, we identified for the first time the lncRNA expression profile of BMSCs from the patients with SONFH. 3720 lncRNAs and 2775 mRNAs were found to be differentially expressed (fold change ≥2, FDR < 0.05). More importantly, the lncRNA expression profile of BMSCs in SONFH was found to be significantly associated with increased adipogenic and decreased osteogenic differentiation. The potential roles of lncRNA RP1-193H18.2, HOTAIR and MALAT1 in the abnormal transdifferentiation between osteogenesis and adipogenesis of BMSCs in SONFH were identified using CNC, ceRNA network and qRT-PCR. Our results present new insights that support further exploration of the abnormal adipogenic and osteogenic transdifferentiation of BMSCs during the occurrence and development of SONFH and open a new field to find molecular targets for SONFH treatment.

## Methods

### Patients and controls

Sixteen patients with SONFH (SONFH group, age range 40–77 years) and sixteen patients with femoral neck fracture (control group, age range 62–77 years) were enrolled from the Department of Orthopedics, the Second Clinical College of Jilin University, China from March 2016 to January 2017. Diagnosis of ONFH was confirmed by preoperative radiographs and magnetic resonance image (MRI) according to the Steinberg or University of Pennsylvania system. Steroid-induced osteonecrosis was defined by a history of taking a mean daily dose of 16.6 mg or an equivalent maximum daily dose of 80 mg of prednisolone within 1 year^49,50^. Femoral head collapse was measured according to the Nishii method^51^. Patients with concurrent congenital diseases, alcohol consumption or tumor-related diseases were excluded. None of the patients were receiving any medications that could affect bone metabolism. The characteristics of the patients in this study are presented in Table 2. The study was approved by the ethics committee of the Second Hospital of Jilin University, China [(2016)50]. We obtained written informed consent from all participants for the use of their specimens and all involved methods were performed in accordance with the approved guidelines of the Ethics Committee of the Second Hospital of Jilin University.

**Table 2:** Characteristics of the patients in this study.

Variable	SONFH (n = 16)	Control (n = 16)	P-value
Age (years)	66.31 ± 8.25	70.69 ± 4.12	0.076
Gender (M/F)	11/5	3/13	—
CRP (mg/dL)	1.31 ± 2.47	2.47 ± 2.1	0.098
ESR (mm/h)	23.19 ± 18.45	26.56 ± 20.02	0.635
BMI (kg/m^2^)	26.23 ± 3.57	24.38 ± 2.72	0.119
Steinberg staging system (VI/V)	4/ 12	—	—
Femoral head collapse (mm)	10.96 ± 7.06	—	—
Harris score	55.19 ± 5.41	—	—

CRP, C-reaction protein; ESR, erythrocyte sedimentation rate; BMI, body mass index.

### BMSC isolation and culture

BMSCs were isolated from the bone marrow of the proximal femur of patients by density gradient centrifugation^52^. The cells were cultured in Dulbecco’s modified Eagle’s medium (DMEM, GIBCO, NY, USA) with 10% fetal bovine serum (GIBCO) at 37 °C in 5% CO_2_. The medium was replaced every 3 days. BMSCs were digested and reseeded in new plates when the culture reached 90% confluence. BMSCs were expanded and used for experiments at passage 4.

### Flow cytometry

Cell cycle analysis was performed using a Cell Cycle Kit (TIANDZ, Beijing, China) according to the manufacturer’s protocol. BMSCs were dissociated and fixed in 70% ethanol at 4 °C for 24 h. The cells were then stained with 50 μg/ml PI and treated with 50 μg/ml RNase A at 37 °C for 30 min and assessed by flow cytometry (Beckman Coulter, Brea, CA, USA). For identification of cell surface markers, BMSCs were incubated with P-phycoerythrin (PE)-conjugated anti-human IgG1 or CD73 or fluorescein isothiocyanate (FITC)-conjugated anti-human IgG1, CD34, CD45, CD90 or CD105 (Miltenyi Biotec, Gladbach, Germany) in 5% CO_2_ for 30 min. All samples were assessed by flow cytometry (BeckmanCoulter, Brea, CA, USA).

### Cell proliferation assay

BMSCs were seeded in 96-well plates at a density of 10^3^ cells/well/100 µL, and proliferation ability was detected using Cell Counting Kit-8 (Dojindo Laboratories, Kumamoto, Japan) according to the manufacturer’s protocol. Cell proliferation was measured by determining the optical density (OD) values at 450 nm.

### Osteogenic and adipogenic differentiation

Osteogenic differentiation was induced by culturing BMSCs in osteogenic medium supplemented with 10%FBS, 0.1 μmol/L dexamethasone, 10 μmol/L β-glycerophosphate, 10 μmol/L glutamine and 50 μg/mL ascorbate (Cyagen Biosciences, Guangzhou, China). Adipogenic differentiation was induced by culturing the BMSCs in adipogenic medium supplemented with 10%FBS, 1 μmol/L dexamethasone, 100 μg/mL 3-isobutyl-1-methylxanthine, 2 μg/L insulin, 1 μmol/L rosiglitazone and 10 μmol/L glutamine (Cyagen).

### Alizarin Red S (ARS), alkaline phosphatase (ALP), and Oil Red O staining and quantification

For ARS staining, BMSCs were fixed in 4% paraformaldehyde and stained with 1% ARS (pH 4.3) for 30 min. After being washed three times with PBS, the stained cells were observed via microscopy (Leica Microsystems, Buffalo Grove, United States). For the quantification assay, ImageJ V1.48 (National Institutes of Mental Health, Bethesda, USA) was used to calculate the proportion of ARS-stained areas.

The ALP staining assay was performed using an Alkaline Phosphatase Color Development Kit (Beyotime, Shanghai, China) according to the manufacturer’s protocol, and the cells were observed via microscopy. ALP activity was detected using an Alkaline Phosphatase Assay Kit (Beyotime). BMSCs were lysed in RIPA buffer (Beyotime) and incubated with p-nitrophenyl phosphate (pNPP, Beyotime) at 37 °C for 30 min. Total protein content was calculated using an Enhanced BCA Protein Assay Kit (Beyotime). The ALP activity was ultimately expressed as nmol/(min*mg).

Oil Red O (Beyotime) staining was used to assess the intracellular lipid accumulation after adipogenic differentiation. Cells were fixed in 4% neutral buffered formalin for 30 min and then washed with 3% isopropanol, incubated with newly filtered Oil Red O staining solution for 1 h and rinsed with double-distilled H_2_O. To obtain quantitative data, isopropyl alcohol was added to stained culture dishes and the OD values were measured at 490 nm. ImageJ was used to calculate the number of cells with Oil Red O staining.

### Immunohistochemical staining

The immunohistochemical staining assay was performed using a SABC immunohistochemical staining kit (Boster Biological Technology, Wuhan, China) according to the manufacturer’s protocol. The BMSCs were fixed in 4% paraformaldehyde for 30 min and treated with 0.5% Triton X-100 (TIANDZ) for 20 min at room temperature. Endogenous peroxidase was quenched by 3% hydrogen peroxide (H2O2) for 20 min. Then BMSCs were incubated with 5% BSA for 30 min at 37 °C and incubated overnight with anti-Osterix antibody (Bioss, Beijing, China), anti-RUNX2 antibody (Boster), anti-PPARγ antibody (Boster,) and anti-CEBPα antibody (Boster). Goat anti-rabbit immunoglobulin G antibodies were used as secondary antibodies. Diaminobenzidine (Gene Tech, Shanghai, China) served as a chromogen and BMSCs were observed by microscopy.

### RNA extraction

Total RNA was extracted using TRIzol Reagent (Invitrogen, Carlsbad, CA, USA) according to the manufacturer’s protocol. RNA quantity and quality were measured by spectrophotometry (Agilent, Santa Clara, CA, USA).

### Microarray and computational analysis

Arraystar Human lncRNA microarray V3 (GPL16956) which covered 26109 mRNAs and 30586 lncRNAs was used for microarray analysis. Six samples (3 SONFH and 3 control) were used for the microarray analysis. The method is basically the same as that described by Liu *et al*.^53^ and the details are shown as following. Sample labeling and array hybridization were performed according to the Agilent One-Color Microarray-Based Gene Expression Analysis protocol (Agilent). After removal of rRNA mRNA was purified from total RNA and transcribed into fluorescent cRNA along the entire length of the transcripts without 3’ bias utilizing a random priming method. The labeled cRNAs were purified using an RNeasy Mini Kit (Qiagen, USA). The concentration and specific activity of the labeled cRNAs (pmol Cy3/μg) were measured by NanoDrop ND-1000. 1 μg of each labeled cRNA was fragmented by adding 5 μl 10× Blocking Agent and 1 μl of 25× Fragmentation Buffer, then heated the mixture at 60 °C for 30 min, and 25 μl of 2× GE hybridization buffer was added to dilute the labeled cRNA. Then, 50 μl of the hybridization solution was dispensed into the gasket of a slide, and the lncRNA expression microarray slide was assembled. The slides were incubated for 17 h at 65 °C in an Agilent Hybridization Oven. The hybridized arrays were washed and scanned in the Agilent DNA Microarray Scanner (part number G2505C). Agilent Feature Extraction software (version 11.0.1.1) was used to analyze acquired array images. Quantile normalization and subsequent data processing were performed with the GeneSpring GX v12.1 software package (Agilent). After quantile normalization of the raw data, lncRNAs and mRNAs that at least 3 out of 6 samples had flags in Present or Marginal (“All Targets Value”) were chosen for further data analysis. Differentially expressed lncRNAs and mRNAs with statistical significance between the two groups were identified when the change in threshold values was >2.0 or < −2.0 fold and when the Benjamini-Hochberg- corrected P- values were <0.05.

### Quantitative real-time polymerase chain reaction (qRT-PCR)

Total RNA was reverse-transcribed to cDNA using PrimeScript RT reagent Kit with gDNA Eraser (TaKaRa, Japan) according to the manufacturer’s instructions. QRT-PCR was performed using FastStart Universal SYBR Green Master (ROX) (Roche, Basel, Germany) in an Applied Biosystems 7500 Fast Real-Time PCR System (Applied Biosystems) according to the manufacturer’s protocol. The data were normalized to the expression of glyceraldehyde-3-phosphate dehydrogenase (GAPDH), and the relative expression levels of each gene were analyzed using the 2^−△△Ct^ method. All experiments were performed in triplicate. The primer sequences used in this study are shown in (Supplementary Table 4).

### Bioinformatics analysis

Hierarchical clustering was performed using Euclidean distance and average linkage clustering base on lncRNA and mRNA expression profiles. Gene ontology (GO) analysis was performed to determine the roles of differentially expressed mRNAs. The GO project provides a controlled vocabulary to describe gene and gene product attributes (http://www.geneontology.org)^54,55^. Fisher’s exact test was used to find the overlap between the differentially expressed mRNAs and the GO annotation list. Pathway analysis of the differentially expressed genes was also performed based on the Kyoto Encyclopedia of Genes and Genomes (KEGG) database^56–58^ (http://www.genome.ad.jp/kegg, version 83.2) to identify the significantly changed pathways. The enrichment P-value of the Pathway ID was determined used Fisher’s exact test. Coding-non-coding gene co-expression (CNC) networks were constructed based on the results of correlation analyses between differentially expressed mRNAs and lncRNAs (Pearson correlation coefficient ≥0.99 or ≤ −0.99).

### Construction of the ceRNA network

We constructed a ceRNA network under the following conditions: (I) six lncRNAs with the largest fold change in the microarray data (3 up-regulated and 3 down-regulated) were used. To enhance data reliability, the lncRNAs that were not recorded in ENCODE were removed; (II) lncRNA-miRNA interactions were predicted by Diana Tools^59^ (http://diana.imis.athena-innovation.gr/DianaTools); (III) Targetscan^60^ (http://www.targetscan.org) and miRDB^61^ (http://www.mirdb.org/miRDB/) were used to predicted miRNA-mRNA interactions.

### Statistical analysis

The data were presented as the mean ± standard deviation. Statistical analyses were performed using SPSS version 20.0 (SPSS Inc., Chicago, IL, USA). Comparisons between groups were performed using unpaired Student’s t-test. Fisher’s exact test was used to evaluate the significance of GO terms and Pathway identifiers enrichment. The Pearson correlation coefficient was used to examine the relationship between lncRNAs and co-expressed mRNAs. The false discovery rate (FDR) controlling was used to correct p-value with Benjamini Hochberg algorithm implemented in R 3.4.1 suite (Lucent Technologies). FDR and P-value less than 0.05 was considered statistically significant.

### Data availability

The datasets generated in the current study are available from the corresponding author upon reasonable request.

## Electronic supplementary material

Supplemental Figures and tables

Dataset 1

Dataset 2
