# Supplementary material for: LncRNA expression profiling of BMSCs in osteonecrosis of the femoral head associated with increased adipogenic and decreased osteogenic differentiation
Source: Sci Rep. 2018 Jun 14;8:9127. doi: 10.1038/s41598-018-27501-2 (PMC6002551; doi:10.1038/s41598-018-27501-2)
Supplement: Supplementary file 1 — Supplemental Figures and tables [file 41598_2018_27501_MOESM1_ESM.pdf]

LncRNA expression profiling of BMSCs in osteonecrosis of the femoral head associated with increased adipogenic and decreased osteogenic differentiation

Qingyu Wang[a,b,c], Qiwei Yang[b,c], Gaoyang Chen[b,c], Zhenwu Du[a,b,c], Ming Ren [a,c], Ao Wang[a], Haiyue Zhao[b,c], Zhaoyan Li[a,c], Guizhen Zhang[*,a,b,c], Yang Song[*,a,c]

a Department of Orthopedics of the Second Hospital of Jilin University, Ziqiang Street 218, Changchun, Jilin, 130041, China

b Research Centre of the Second Hospital of Jilin University, Ziqiang Street 218, Changchun, Jilin, 130041, China

c The Engineering Research Centre of Molecular Diagnosis and Cell Treatment for Metabolic Bone Diseases of Jilin Province, Ziqiang Street 218, Changchun, Jilin, 130041, China

[*] Corresponding Author:

Guizhen Zhang, M.D. and Ph.D., Department of Orthopedics of the Second Hospital of Jilin University, Ziqiang Street 218, Changchun, Jilin, 130041, China. Tel.: 011-86 431-88796301, Fax: 011-86 431-88796301, E-mail address: zhangguizhenjlu@163.com;

Yang Song, M.D., Department of Orthopedics of the Second Hospital of Jilin University, Ziqiang Street 218, Changchun, Jilin, 130041, China. Tel.: 011-86 431-88796890, Fax: 011-86 431-88796890, E-mail address: songyangjlu@foxmail.com.

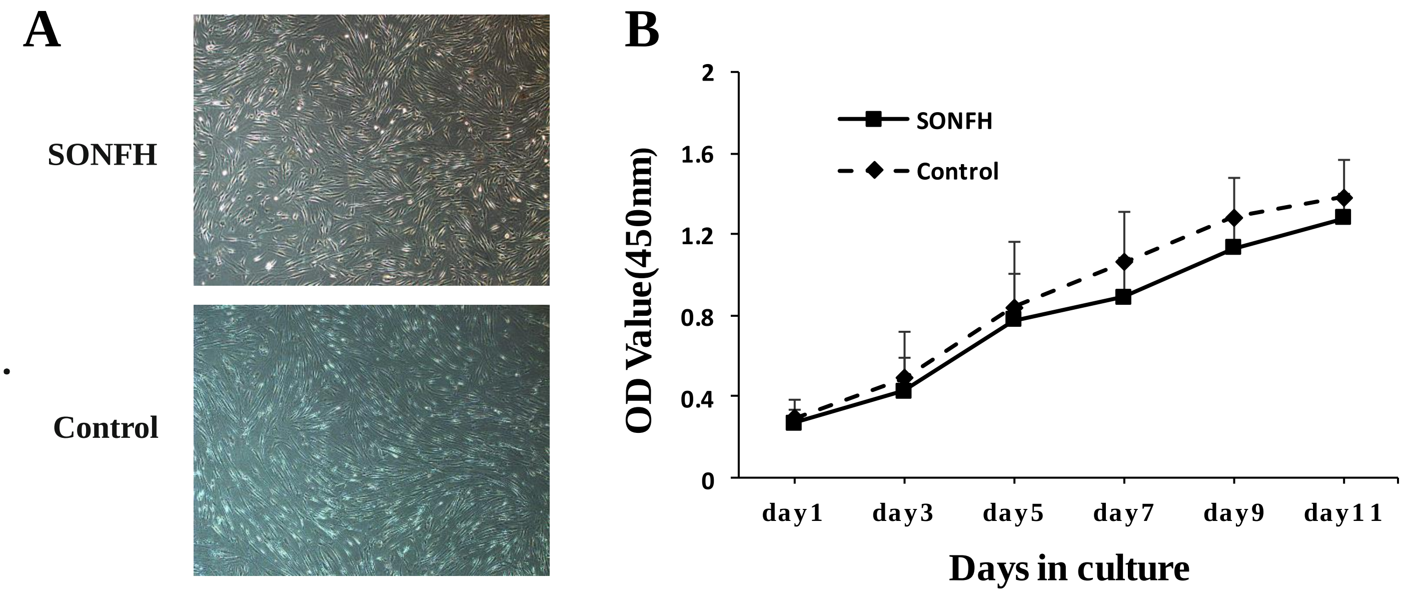

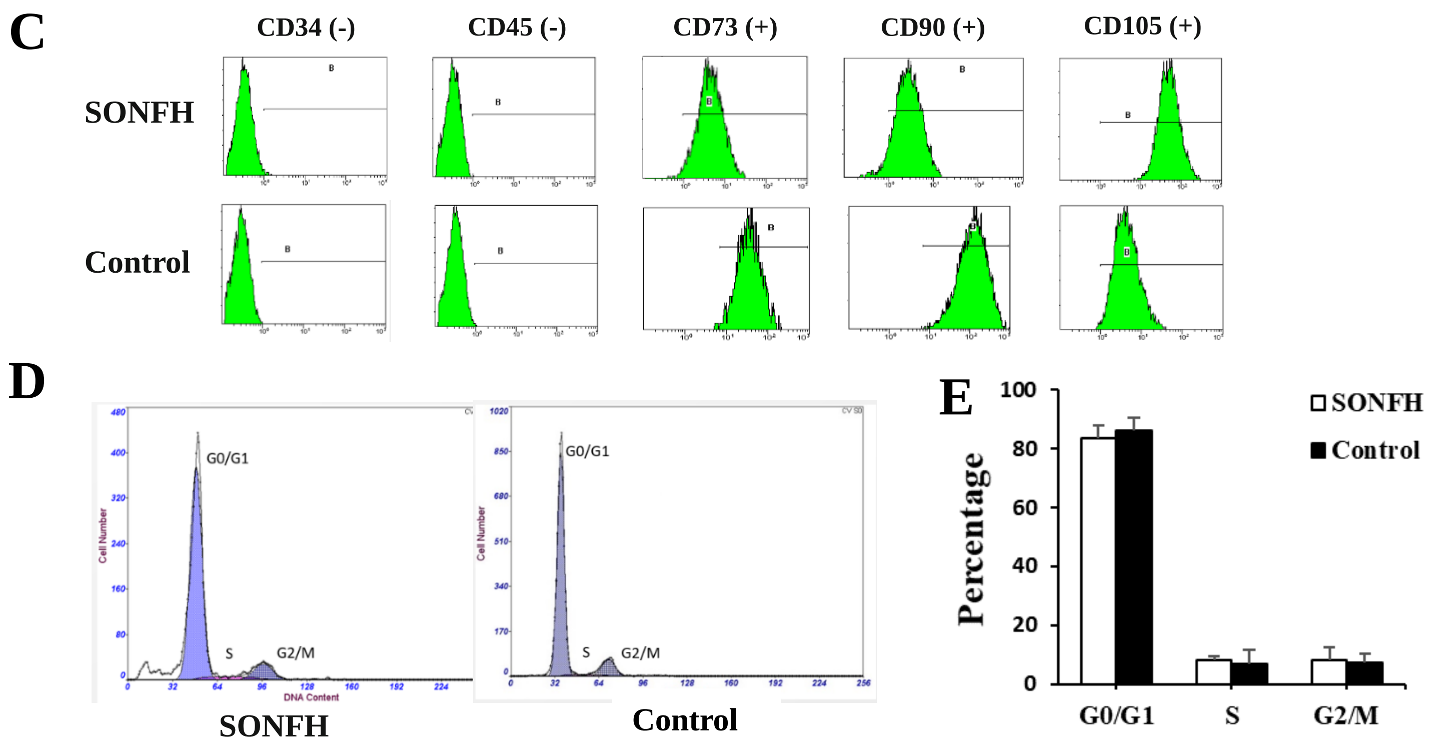

Supplemental Figure 1. The phenotype and proliferation of BMSCs showed no significant differences between the SONFH group and control group. (**A**) The morphologies of BMSCs from SONFH group and control group were identical. (**B**) The proliferation capacities of BMSCs from the SONFH group (n=12) and control group (n=12) had no significantly differences when cultured in growth medium for 1 to 11 days. (**C**) BMSCs from the SONFH group (n=7) and control group (n=7) were both positive for CD90, CD73, and CD105 and negative for CD34 and CD45, indicating a typical MSC phenotype. (**D**, **E**) The cell cycle distribution of the BMSCs in both groups had no significantly differences (n=6 in each group).

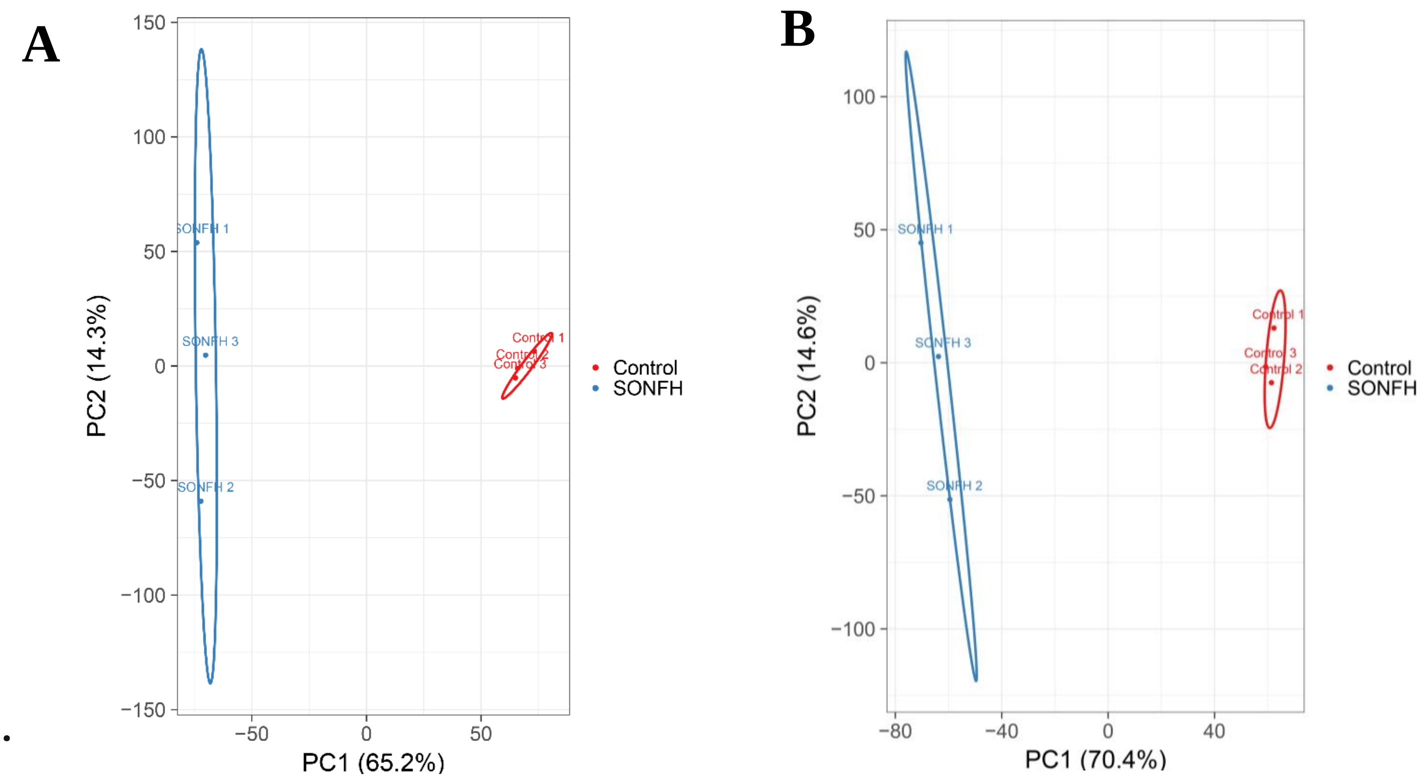

Supplemental Figure 2. PCA analysis of lncRNA(A) and mRNA(B) microarray data.

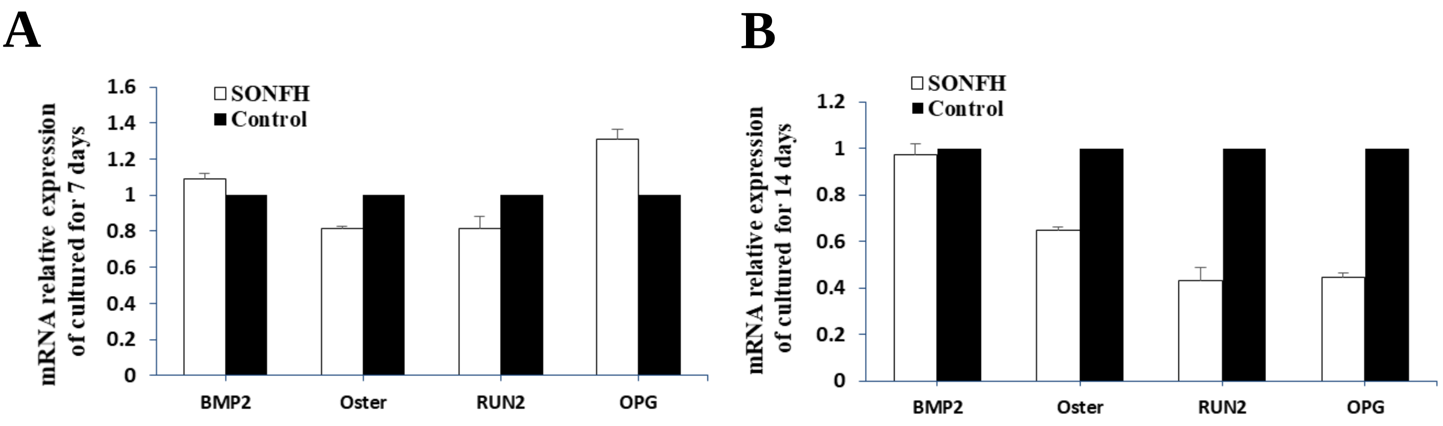

Supplemental Figure 3. The basal expression level of osteogenic markers (RUNX2, Osterix, BMP2, OPG) in BMSCs without osteogenic induction from SONFH group (n=16) and control group (n=16).

Supplemental Table 2. The characteristics of lncRNAs and mRNAs for validation

	Sequence name	Gene Symbol	Expression	RNA length	Chromosome	Stand	Start	End
lncRNA	ENST00000437067	RP1-67K17.3	up	349	chr6	+	143069579	143074896
	ENST00000580645	CTD-2006O16.2	up	464	chr18	+	22882089	22882920
	NR_102430	OGFR-AS1	up	684	chr20	-	61431978	61436939
	NR_038340	LOC100505817	up	1181	chr18	+	70992175	71017124
	NR_047517	HOTAIR	up	2370	chr12	-	54356091	54368740
	ENST00000588041	RP1-193H18.2	down	2504	chr17	+	67547498	67550002
	NR_002819	MALAT1	down	8758	chr11	+	65265232	65273983
	NR_026812	RUNX1-IT1	down	1502	chr21	-	36410232	36411723
	ENST00000517495	CTD-3080F16.3	down	255	chr8	-	41132633	41134522
	ENST00000607490	XXbac-BPGBPG55C20.3	down	540	chr6	-	58228886	58229426
mRNA	NM_016639	TNFRSF12A	up	1048	chr16	+	3070312	3072383
	NM_030967	KRTAP1-1	up	903	chr17	-	39196810	39197713
	NM_012242	DKK1	up	1815	chr10	+	54074040	54077417
	NM_002521	NPPB	up	708	chr1	-	11917520	11918992
	NM_032598	SPATA22	up	1479	chr17	-	3343305	3375041
	NM_015461	ZNF521	down	4985	chr18	-	22641887	22932214
	NM_003012	SFRP1	down	4482	chr8	-	41119475	41166990
	NM_006734	HIVEP2	down	9732	chr6	-	143072603	143266338
	NM_002825	PTN	down	1558	chr7	-	136912091	137028546
	NM_144715	EFHB	down	2857	chr3	-	19920965	19975706

Supplemental Table 4. Primers for mRNAs and LncRNAs in real-time RT-PCR analysis

**Gene**	**Primer sequences**	
RUNX2	F: 5’- AGATGGGACTGTGGTTACTG-3’	R: 5’- GTAGCTACTTGGGGAGGATT-3’
BMP2	F: 5’- GGAATGACTGGATTGTGGCT-3’	R: 5’- TGAGTTCTGTCGGGACACAG-3’
OPG	F: 5’- CACTCCTCGCCCTATTGGC-3’	R: 5’- CCCTCCTGCTTGGACACAAAG-3’
PPARγ	F: 5’- GAGCCCAAGTTTGAGTTTGC-3’	R: 5’- CTGTGAGGACTCAGGGTGGT-3’
C/EBPα	F: 5’- GGGCAAGGCCAAGAAGTC -3’	R: 5’- TTGTCACTGGTCAGCTCCAG -3’
Adipsin	F: 5’- TGCTACAGCTGTCGGAGAAG -3’	R: 5’- ATGACTTCATTGCTCGGGAC -3’
EFHB	F: 5’- ATCAATGCAATTGTAGGAGCCATTC -3’	R: 5’- TGAATATGCACTACCTTCTTCACCA -3’
PTN	F: 5’- GTAATTGAGTCAAAGGCAGGATCAG -3’	R: 5’- CTCTCTCCACTTTGGATTTTCCTCT -3’
SFRP1	F: 5’- CTACATTTTGCAACTTGTTGGTGTG -3’	R: 5’- GACAGAAAGAAACTACTCTGGACCT -3’
HIVEP2	F: 5’- TAACATACAGCCACTAGATTCCCAG -3’	R: 5’- ACGCTTCCTGTTATCCTCATATCAA-3’
ZNF521	F: 5’- CTTTTAATCATGCCCCTCTGTCTGT-3’	R: 5’- TCCGGTAGTCCACATAATAATGGAAA-3’
KRTAP1-1	F: 5’- CTGAACTCCAGTACCTATAACTGGG-3’	R: 5’- TCTTTTCACATTGTAGGACTTTGGC-3’
SPATA22	F: 5’- AAAGACTGTGCGTTGATACCAAACT-3’	R: 5’-ATTTAGGCTTCGCTTCATTTCCTTC-3’
NPPB	F: 5’- TTCTGATTCCACAAGGGGCTTTTTC-3’	R: 5’- TTGTGCTCAAAGGTAAGAAACCATC-3’
TNFRSF12A	F: 5’- AACAGAAAGGGAGCCTCACG -3’	R: 5’- GTGGGGCCTAGTGTCAAGTC -3’
DKK1	F: 5’- ACACTTCTGGTCCAAGATCTGTAAA-3’	R: 5’- ACGCTGGAATATTTCTAGTCCATGA-3’
GAPDH	F: 5’- CGGACCAATACGACCAAATCCG-3’	R: 5’- AGCCACATCGCTCAGACACC-3’
RP1-193H18.2	F: 5’- AGGTGGATTTCTTCAGCTTTCTTTG-3’	R: 5’- AGGTAAGAAAACGTGTTGTTGTGTT-3’
XXbac-BPGBPG55C20.3	F: 5’- ACAGAAGGTACTGACTTATTTGGTGT-3’	R: 5’- GACTGAAGTGAGGGATTTAGGAGAA-3’
MALAT1	F: 5’- TGCAGTTTTAAGCAGTCGTATTTGT-3’	R: 5’- GCTTGTTTGGAATGTTTCTTGTCAC-3’
CTD-3080F16.3	F: 5’- GAATCATAAAGCTCTGGGTAGGGAA-3’	R: 5’- AACTGGAAGAGACGTTAAATCTGGT-3’
RUNX1-IT1	F: 5’- CTAGCAGACACTTCTCCTAATCCTC-3’	R: 5’- ATATACATTGCCCTGCCTAATGAGT-3’

OGFR-AS1	F: 5’- GGTAACAAAAGTAACAAGCGGAGG-3’	R: 5’- CTTTATGTAATGCACACTCCACCAT-3’
LOC100505817	F: 5’- CAAGGAGAAACTGAAGAGCAAGAAG-3’	R: 5’- CTCAAACTCTTTCAGGCACATAAGG-3’
HOTAIR	F: 5’- AAAAAGCTTGGGTTATAGGAAAGCC-3’	R: 5’- GCTTCTATGTTCCTCTCAAATTCCG-3’
RP1-67K17.3	F: 5’- TTCGGTGCCAAGTAGAATAATTTCC-3’	R: 5’- CAGCCCAGACTTTATATGCAACAAT-3’
CTD-2006O16.2	F: 5’- TTTGTTTATAGATGCTGGGAAAGGC-3’	R: 5’- ATTACTCCTGCTTCTTTACTGGTGT-3’

EFHB, EF-hand domain family member B; PTN, pleiotrophin; SFRP1, secreted frizzled related protein 1; ZNF521,zinc finger protein 521; KRTAP1-1 ,keratin associated protein 1-1;SPATA22, spermatogenesis associated 22; NPPB, natriuretic peptide B; TNFRSF12A, TNF receptor superfamily member 12a; DKK1,dickkopf WNT signaling pathway inhibitor 1. GAPDH, glyceraldehyde-3-phosphate dehydrogenase. MALAT1, Metastasis-associated lung adenocarcinoma transcript 1. RUNX1-IT1, RUNX1 intronic transcript 1. OGFR-AS1, OGFR antisense RNA 1. HOTAIR, HOX transcript antisense RNA
